# Towards a Circular Economy: Study of the Mechanical, Thermal, and Electrical Properties of Recycled Polypropylene and Their Composite Materials

**DOI:** 10.3390/polym14245482

**Published:** 2022-12-14

**Authors:** Tongsai Jamnongkan, Nitchanan Intraramongkol, Wesarach Samoechip, Pranut Potiyaraj, Rattanaphol Mongkholrattanasit, Porntip Jamnongkan, Piyada Wongwachirakorn, Masataka Sugimoto, Hiroshi Ito, Chih-Feng Huang

**Affiliations:** 1 Department of Fundamental Science and Physical Education, Faculty of Science at Sriracha, Kasetsart University, Chonburi 20230, Thailand; 2 Department of Materials Science, Faculty of Science, Chulalongkorn University, Bangkok 10330, Thailand; 3 Faculty of Industrial Textiles and Fashion Design, Rajamangala University of Technology Phra Nakhon, Bangkok 10110, Thailand; 4 Department of Public Health and Environment, Saensuk Municipality, Chonburi 20130, Thailand; 5 Department of Environmental Science, Faculty of Science and Technology, Pibulsongkarm Rajabhat University, Phitsanulok 65000, Thailand; 6 Graduated School of Organic Materials Science, Faculty of Engineering, Yamagata University, Yonezawa 32000, Yamagata, Japan; 7 Department of Chemical Engineering, i-Center for Advanced Science and Technology (iCAST), National Chung Hsing University, Taichung 40227, Taiwan

**Keywords:** polypropylene, plastic waste, mechanical properties, carbon black, sustainability

## Abstract

This research focuses on the mechanical properties of polypropylene (PP) blended with recycled PP (rPP) at various concentrations. The rPP can be added at up to 40 wt% into the PP matrix without significantly affecting the mechanical properties. MFI of blended PP increased with increasing rPP content. Modulus and tensile strength of PP slightly decreased with increased rPP content, while the elongation at break increased to up to 30.68% with a 40 wt% increase in rPP content. This is probably caused by the interfacial adhesion of PP and rPP during the blending process. The electrical conductivity of materials was improved by adding carbon black into the rPP matrices. It has a significant effect on the mechanical and electrical properties of the composites. Stress-strain curves of composites changed from ductile to brittle behaviors. This could be caused by the poor interfacial interaction between rPP and carbon black. FTIR spectra indicate that carbon black did not have any chemical reactions with the PP chains. The obtained composites exhibited good performance in the electrical properties tested. Finally, DSC results showed that rPP and carbon black could act as nucleating agents and thus increase the degree of crystallinity of PP.

## 1. Introduction

In recent times, the production of petroleum-based plastic materials has rapidly increased because they exhibit several advantages for various uses over other materials such as a light weight, high flexibility, resilience, resistance to corrosion, excellent chemical resistance, transparency, and ease of processing [1,2,3,4]. It is forecasted that the global annual production of plastic will grow further due to the continued expansion of world population, and therefore the demand for consumables will also increase. Thus, waste management of plastic post-consumption is very important [5,6]. It is well-known that plastic waste has numerous damaging environmental effects, including increased energy consumption and greenhouse gas exhaustion [7,8,9]. Particularly, fossil-based plastics such as polyethylene (PE), polypropylene (PP), polystyrene (PS), polyethylene terephthalate (PET), and many others are used for daily purposes which increases the amounts being disposed of in landfills day by day [10,11,12,13,14,15,16]. In consequence, it has been a challenge to establish a cost-effective method to solve these problems. One effective approach could be the use of natural resources, called biomaterials, to replace their corresponding functional products [17]. Upcycling or recycling plastic waste based on the circular economy (CE) concept is an effective alternative to manage and reduce the effects of plastic waste on the environment [18,19]. This concept could be managed waste from the plastic production and consumption system by imported and consumed as raw materials for recycling, reuse, reduction and repairing or refurbishing in the process of product production. Recently, the concept of CE has received great attention from several scientists across the world because it has directly responded to one of the seventeen targets of the Sustainable Development Goals (SDGs). CE is a manufacturing method for waste management that allows the remanufacturing, reprocessing, and recycling of materials in the production process [20,21]. Therefore, the recycling method not only reduces poisonous materials and waste pollution on the environment compared to landfilling and incineration [22,23,24], but can also create new products with good mechanical and physical properties. However, it is not easy to recycle these materials because they have been combined with different types of plastics and additives during processing [25]. In general, the mechanical properties of recycled plastics do not remain the same because of several parameters such as degradation from heat, mechanical stress, and oxidation during reprocessing. Recently, several research groups have studied and focused on the influence of multiple recycling cycles on the quality of the reprocessed plastics. For example, Canevarolo [26] reported the thermo-mechanical properties of PP when subjected to multi-extrusion with different processing conditions and screw profiles. They found that the material was reprocessed five times and found that the PP chain scission process occurred during multiple processes, particularly at higher molecular weights of PP. Jin et al. [27] have investigated and reported the effects of the recycling process of low-density polyethylene (LDPE) on its mechanical properties. They found that LDPE could be extruded for up to 40 times without significantly changing its processability and long-time mechanical properties. Tominaga and his colleagues worked on the mechanical properties of recycled PP and found that they are closely related to the inner structure of materials [28]. They also mentioned that the molecular weight of recycled PP did not change significantly during the recycling process, and they were able to improve its mechanical properties and durability by adopting an appropriate molding condition of the mechanical recycling process. In Thailand, it was also found that the demand and production of PP have increased tremendously due to its applications in various areas, which has caused an increase in PP waste in landfills. PP is a semicrystalline thermoplastic that can be remelted and used again, and also has a low cost, processability, and excellent chemical stability [29]. Over last decade, it has been attempted to solve the problem of recycled plastics through several methods, both physical (i.e., blending, reprecipitation, composite, etc. [30,31,32,33,34,35]) and chemical (such as reaction processes and catalytic cracking methods [36,37,38,39]), leading to products ready to be used in specific applications. The easiest way is to recycle the material directly by a physical process such as blending it with the virgin material. As we have mentioned earlier, it is still difficult to keep consistency of mechanical properties and performance of raw materials during the recycling of post-consumer materials. In addition, the idea of recycling as any technology that converts post-use plastics into specialty polymers or new materials has been increasingly recognized as a potential solution to the recycling of plastic waste. Therefore, the performance of value-added specialty polymers should be paramount concerns. The electrical conductivity of a polymer is one of the important properties that have recently attracted attention from researchers because of its relevance to various practical fields, such as electronic devices and sensors [40,41,42]. Carbon black has attracted attention due to its numerous desirable properties, such as good electrical conductivity, low cost, and good thermal and chemical stability [43,44]. Accordingly, we have chosen to use carbon black as a conductive filler within rPP matrices. Our aim is to study the reprocessing of problematic industrial PP scrap or rPP by using the melt-extrusion process. The effects of the concentrations of rPP on their mechanical properties were investigated. In addition, the chemical, mechanical, thermal, and surface resistivity properties of the prepared rPP/carbon black composites were also examined. This research will advance the knowledge and help to promote the use of rPP in some material engineering applications.

## 2. Materials and Methods

### 2.1. Materials

Commercial-grade virgin polypropylene (vPP), 1100NK grade, was purchased from IRPC Co., Ltd., Rayong, Thailand. Recycled PP (rPP) was obtained from Panich Parts and Mold Co., Ltd., Chonburi, Thailand. Distilled glycerol monostearate (DMG) and carbon black were received from Suntor Chemical Co., Ltd., Samutprakarn, Thailand. Other chemical agents were of analytical-grade purity and used as received.

### 2.2. Preparation of vPP/rPP Blends

At the first stage, rPP was sorted and crushed into 0.5 cm of granular form by using a pulverizer milling machine (Siamlab, Nonthaburi, Thailand) for enhancing the surface area of the material. After that, it was dried at a temperature of 80 °C for 24 h in a hot air oven to remove the moisture content. Different ratios of vPP/rPP blends were prepared by the extrusion method as follows. rPP and vPP pellets were first dried at 80 °C for 24 h and then the different ratios of rPP/vPP were fed into a lab-scale, twin-screw extruder (Thermo PRISM, Bangkok, Thailand), with a screw diameter of 28 mm and a length/diameter ratio of 25:1, to mix the compounds. During the extrusion process, the fed temperature in the first zone of the extruder and the die temperature were set to 160 and 180 °C, respectively. The extruder temperature profile was set at 170 °C with a screw speed of 40–50 rpm. The extrudate was cooled down in a water bath at a temperature of 30–35 °C. Then, the extrudate was cut into pellets nominally 3 mm long by using a pelletizer. After extrusion, the vPP/rPP compounds were dried and indirectly heated for dehumidifying in the oven at a temperature of 80 °C for 4 h, and then stored in moisture-barrier bags before injection molding. An injection molding machine (Model SG50M, Sumitomo, Tokyo, Japan) was used to fabricate the specimens for the mechanical test. This machine had a single screw of 40 mm in diameter with a length/diameter ratio of 18:1. The temperature of the feeding, compression, and metering sections were maintained at 160, 170, and 175 °C, respectively. During the injection process, the holding step was set to be three steps with pressure and time as follows: (i) 90.0 bar for 3 s, (ii) 95.0 bar for 5 s, and (iii) 90.0 bar for 3 s. Mold temperature was maintained at 40 °C, which was controlled by water cooling. In this paper, the recycling PP samples are referred to as vPP, rPP20, rPP30, and rPP40, corresponding to the virgin PP and the PP blended with rPP at the concentrations of 20, 30, and 40 wt%, respectively. The melt flow index (MFI) of all blended samples was determined with the melt flow index instrument (Kayeness, Model 7053, Morgantown, PA, USA) in accordance with the ASTM D1238 standard. The same sample was tested at least five times at 230 °C with a load of 2.10 kg. The MFI was calculated by using the following equation:

MFI (g/10 min) = 600 m/t

where m and t refer to the average weight of extrudates and the time of extrudate in seconds, respectively.

In addition, rPP30 was chosen as a representative of recycled PP composited with carbon black at the concentration of 40 wt%. This composite was prepared through the same procedure for vPP/rPP blends preparation, as follows: rPP30 was milled and mixed with carbon black and DMG at the concentration of 40 wt% and 0.5 wt%, respectively, by using a high-speed mixer machine. Then, the mixture powder was transferred to the twin screw extruder. During the extrusion process, the temperature in the mixing chamber was set to a range of 160–180 °C and the screw rotation rate was maintained at 210 rpm. In this process, polyethylene (PE) wax was used as a plasticizer to improve the processibility of composites for comparison. After obtaining the composite pellets, the specimens for mechanical testing were fabricated using an injection molding machine following ASTM D638 [45]. In this study, the samples are referred to with codes rPP30CBW and rPP30CBNW, corresponding to the rPP30 composited with 40 wt% of carbon black with and without PE wax, respectively.

### 2.3. Tensile Test

Tensile tests of all the samples were performed on the universal testing machine (Model 5560, Instron, MA, USA). A load cell of 25 kN was employed for testing all the samples. All the specimens were fabricated to a rectangular shape (30 × 10 × 0.4 mm^3^), according to the ASTM D638 [45], by using an injection molding machine. Crosshead speed and gauge length were set at 5 mm/min and 25 mm, respectively. At least five samples were tested in each sample and the average of results were reported.

### 2.4. FTIR-ATR Analysis

FTIR was performed to examine the presence of chemical reactions within the structure of compounding molecules. An FTIR spectrometer (Invenio, Bruker, MA, USA) in ATR mode equipped with a diamond crystal was used for the tests. All the spectra were recorded in transmittance mode with 4 cm^−1^ resolutions in the wavenumber range of 4000–400 cm^−1^ under ambient conditions. 

### 2.5. Surface Resistivity Test

Electrostatic discharge is one of the key issues that cause losses in the properties of products containing sensitive electronics during production, storage, and transportation. Therefore, the surface resistivity of rPP30 composited with carbon black was measured at room temperature using a probe digital multimeter (Model KS-385B, Kingsom, Shenzhen, China), according to ASTM D257 [46]. The specimen of vPP was also investigated for comparison. The experimental procedure was as follows: two copper electrodes were pressed on the surface of the composites at a distance of 1.5 cm from each other.

### 2.6. Differential Scanning Calorimetry Test

The crystallization behavior and melting characteristics of vPP, rPP30, and its composites were analyzed by using a differential scanning calorimetry (DSC) technique (Model DSC 200 F3, Netzsch, Selb, Wunsiedel, Germany). All the samples were in a sealed aluminum crucible and each sample used was approximately 5 mg. DSC analysis was run at a temperature range from 25 to 200 °C, with heating and cooling rates of 10 °C/min. All runs were carried out under inert atmosphere with a nitrogen flow of 50 mL/min to prevent the thermal degradation of samples. After that, the degree of crystallinity (χ_c_, %) was determined from the melting enthalpy values using the following equation:

$$\chi_{c}/\%=\frac{\Delta H_{m}}{w\times \Delta H_{m}^{{}^{\circ}}}\times 100$$

where $\Delta H_{m}$, *w*, and $\Delta H_{m}^{{}^{\circ}}$ are the melting enthalpy of the specimens, the weight fraction of filler in PP composites, and the enthalpy value for a theoretically 100% crystalline PP (207 J/g) [47], respectively.

## 3. Results and Discussion

### 3.1. Feasibility of the Obtained vPP/rPP Blends

**Figure 1:**
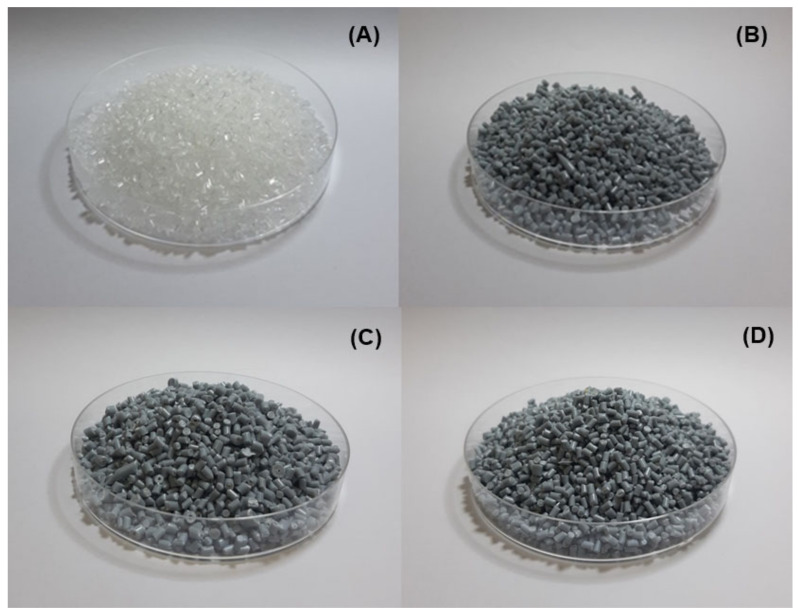
Feasibility images of different compounds. (**A**) vPP, (**B**) rPP20, (**C**) rPP30, and (**D**) rPP40.

**Figure 2:**
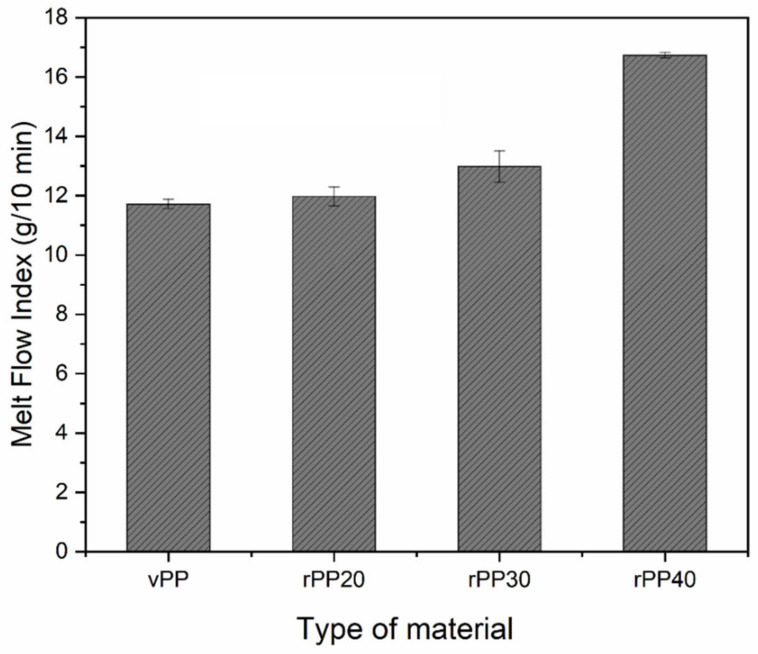
Melt flow index of all prepared samples.

Figure 1 shows optical images of the compound pellets of vPP and vPP blended with rPP at different concentrations. The images show that the extrusion process can produce homogeneous pellets composed of vPP and its blends, with roughly the same external surface in the four samples. Furthermore, the images suggest that the color of the vPP/rPP compounds became gray in the case of the vPP containing rPP when compared to those without rPP. Additionally, the shade of gray color of vPP/rPP compounds is directly proportional to the rPP content within the compounds, as can be seen in Figure 1. This result could indicate that the rPP content will affect mechanical properties. Therefore, it is worthwhile to further examine the effects of rPP content on mechanical properties. We begin our examination of the effect of rPP concentration on the melt flow behavior (results shown in Figure 2). The MFI of PP blends is proportional to the amount of rPP concentration. The MFI of blended vPP increased with increasing rPP content. It was found that vPP and its blend with 20, 30, and 40 wt% of rPP content could be extruded evidently and their MFI values were 11.72, 11.98, 12.98, and 16.74 g/10 min, respectively. Obviously, rPP has extremely affected the melt flow property of PP. This suggested that the high MFI of PP-blended compounds led to poor interfacial adhesion between the filler particles and matrices, which is in agreement with the research results of Xu et al. [48].

### 3.2. Tensile Test

**Figure 3:**
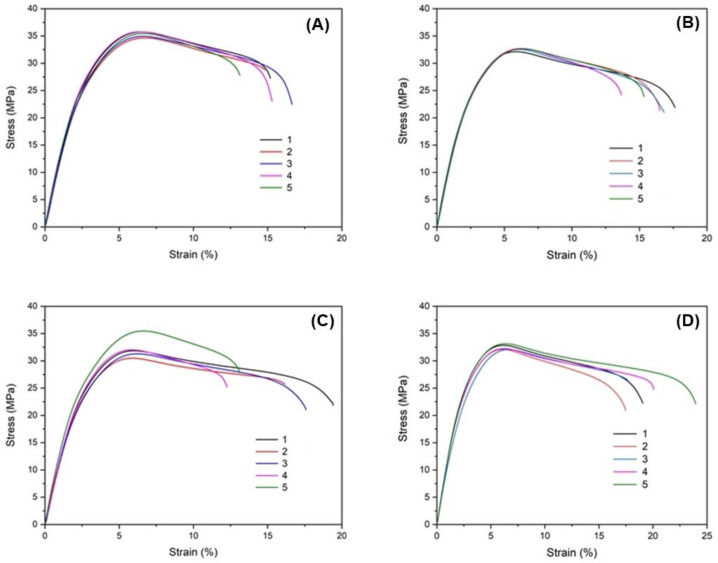
Tensile stress-strain curves of vPP/rPP blended with different rPP concentrations. (**A**) vPP, (**B**) rPP20, (**C**) rPP30, and (**D**) rPP40. The number of each curve indicates the testing numbers.

**Figure 4:**
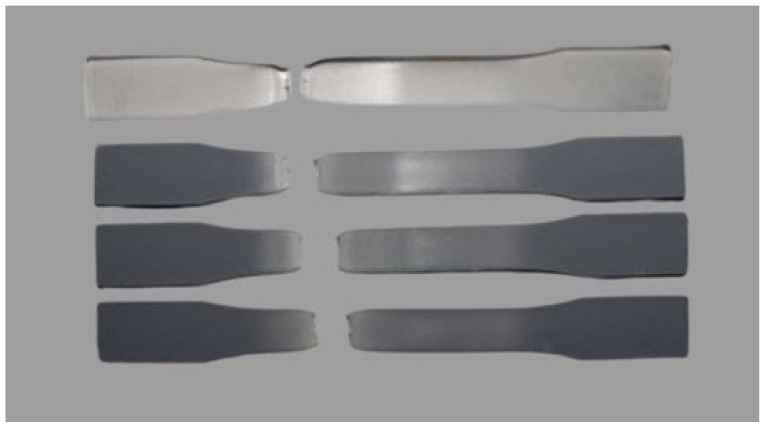
Optical images of all specimens after tensile test at room temperature.

The fracture profile of tensile stress-strain curves and optical feasibility fracture for all specimens were obtained and are illustrated in Figure 3 and Figure 4, respectively. We found that all samples exhibited the fracture ductility behavior.

**Figure 5:**
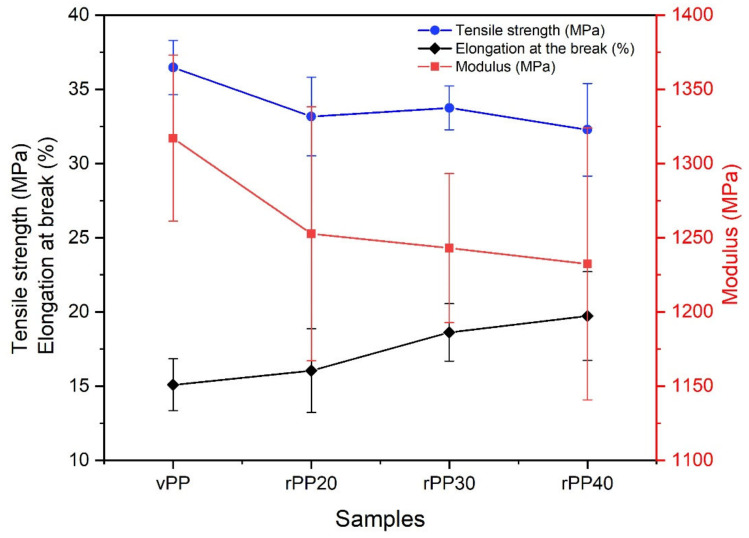
Effect of varying rPP contents on the mechanical properties of PP blends.

The tensile strength, modulus, and elongation at the break of all samples were obtained and presented in Figure 5 and Table 1. In general, the interfacial adhesion between the fillers and the matrices has directly affected the mechanical properties of blends and composite materials [49,50]. We found that the modulus and tensile strength of the vPP/rPP blends slightly decreased with increasing rPP concentration. In addition, the elongation at the breaks of all samples increased with increasing rPP concentration, as enumerated in Table 1. This phenomenon is probably caused by the interfacial adhesion of vPP and rPP during the melt extrusion process.

In Figure 5, we found that vPP exhibited the highest modulus (1317 MPa) and tensile strength (36.47 MPa), while it has the lowest elongation at the break (15.09%) among the four specimens. This result has the same tendency of earlier reports [51,52,53]. In this study, however, we found that the mechanical properties of the recycled PP samples are not insignificantly different when compared with the virgin PP. The experimental results seem to indicate that rPP can be added to a blend with vPP up to a 40 wt% concentration without significantly affecting the mechanical properties.

**Figure 6:**
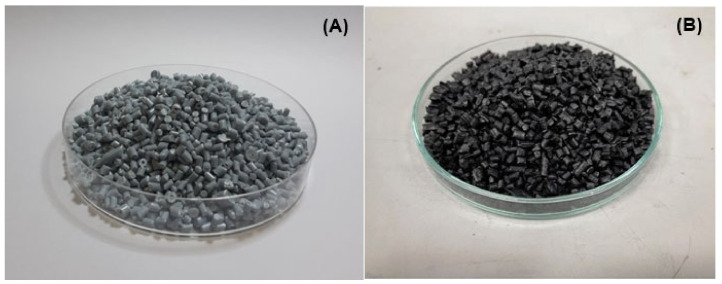
Optical images of pellets of compound (**A**) rPP30 and (**B**) rPP30 composited with carbon black.

**Figure 7:**
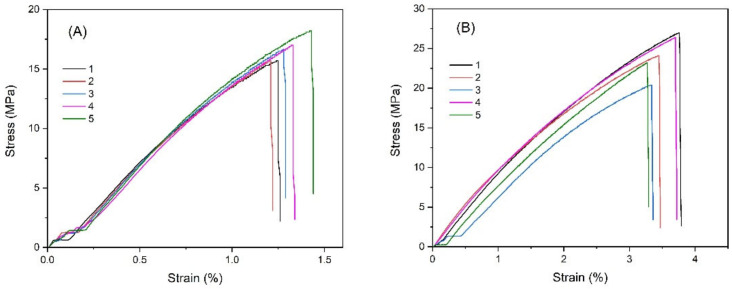
The stress-strain curves of (**A**) rPP30CBNW and (**B**) rPP30CBW composites. The number of each curve indicates the testing numbers.

To develop innovative materials and increase the value addition of PP waste, we attempted to improve the electrical conductivity properties of the rPPs by compositing with carbon black. rPP30 was chosen as a representative of vPP/rPP blends for mixing with carbon black at the concentration of 40 wt%. We successfully compounded these composite materials by using the melt-extrusion process. It was found that the color of the obtained composite compound changed from gray to black, as depicted in Figure 6, coinciding with the natural color of carbon black. Then, we continued to investigate their mechanical properties and found that the stress-strain curves of all composite samples exhibited brittle profile curves, as illustrated in Figure 7.

Mechanical properties such as modulus, tensile strength, and elongation at the break of these composite materials were analyzed and calculated from the results in Figure 7. We found that carbon black significantly affected the modulus, tensile strength, and elongation at the break of composite materials, as shown in detail in Table 2.

**Figure 8:**
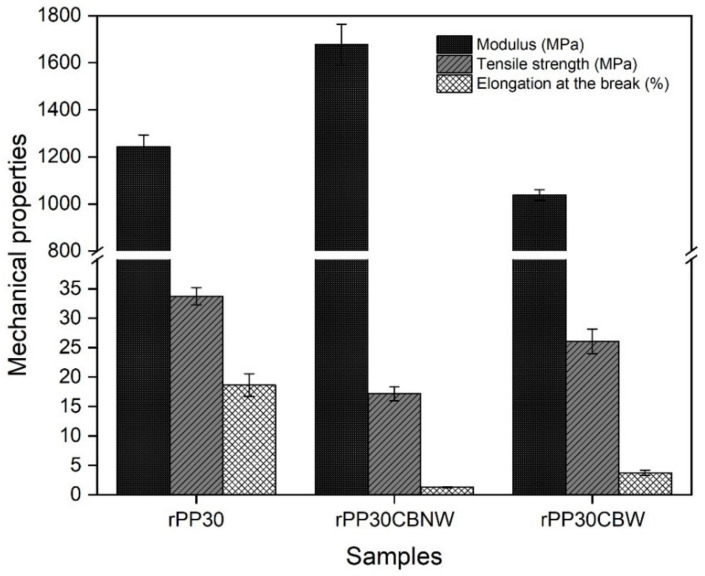
Mechanical properties of the rPP30 and the obtained rPP30 composited with and without the plasticizer content.

Figure 8 shows the mechanical properties of all rPP30/carbon black composite materials compared to rPP30. The rPP30CBNW displayed the highest modulus (1676 MPa) as well as the lowest tensile strength (17.16 MPa) and elongation at the break (1.27%) among the three composite materials. The elongation at the break of rPP30CBW was greater than that of rPP30CBNW. This might be attributed to the addition of a plasticizer that makes the polymer chain move easily, thereby causing the material to have less stiffness. Thus, the materials will have lower modulus and higher percentage of elongation at the break than those composites without the plasticizer [54,55]. Additionally, the interfacial adhesion between filler particles and matrices is also a key parameter that affects their mechanical properties [56], as described earlier.

### 3.3. FTIR–ATR Analysis

**Figure 9:**
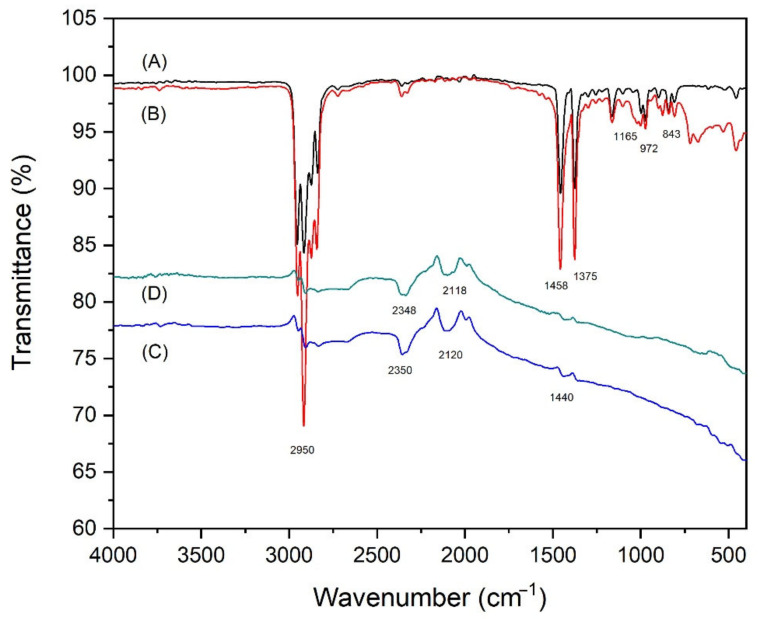
FTIR spectra of (**A**) vPP, (**B**) rPP30, (**C**) rPP30CBW, and (**D**) rPP30CBNW composites.

FTIR was employed to analyze the chemical interaction of the vPP/rPP blends and rPP30/carbon black composites. We began by investigating the IR spectra of rPP in comparison to vPP and the result was depicted in the Supplementary Material, as shown in Figure S1. Figure 9 depicts the FTIR spectrum of rPP30 and rPP30 composited with carbon black samples. IR spectra of the obtained composites and vPP were then compared. We found that the IR spectra of vPP and rPP30 show almost the same characteristic peaks of polypropylene, as shown in spectra A and B, respectively. A strong band around 2950 cm^−1^ revealed the -CH_3_ asymmetrical stretching vibrations of the surface of PP [57]. Moreover, two sharp signals at 1375 and 1458 cm^−1^ indicate the presence of C-H bonds in the chemical structure [58]. In addition, we also found the identified characteristic peaks of the partially crystalline PP such as the transmittance peaks occurring at around wavelengths 1165, 972, and 843 cm^−1^, corresponding to the asymmetrical -C-H stretching vibration, -CH_3_C-C rocking, and -CH_2_- rocking vibration of polypropylene, respectively [57,59].

The IR spectra of rPP30CBW and rPP30CBNW similarly indicated the presence of the functional group of carbon black within the matrices of polypropylene, as illustrated in spectra C and D, respectively. The spectra contain information about both the organic and inorganic parts of the materials. A peak at around 2120 cm^−1^ reveals the presence of a triple bond between C-C atoms (alkynes group) with stretching vibration, as reported in a previous publication [60]. The peak at around 2348–2350 cm^−1^ is ascribed to asymmetrical stretching vibrations of carbon dioxide molecules. Its presence could be associated with the porous nature of carbon black samples [61]. In addition, we found that the weak peaks around 840 cm^−1^ is attributed to out-of-plane deformation vibrations of C-H groups in aromatic structures [62]. The spectrum displays all the characteristic peaks of both carbon black and polypropylene. Thus, we deduced that no chemical bonding occurred between the carbon black and rPP30 molecules. Even though the spectra of the composites contain all the characteristic absorption bands of the polypropylene molecule (i.e., -CH_3_, -CH_2_, -CH, -C-C- stretching), but they are slightly shifted to lower wavelengths and exhibit predominantly low intensities. This phenomenon is probably caused by the effect of the intermolecular interaction between polypropylene chains and carbon black particles.

### 3.4. Surface Resistivity Test

**Figure 10:**
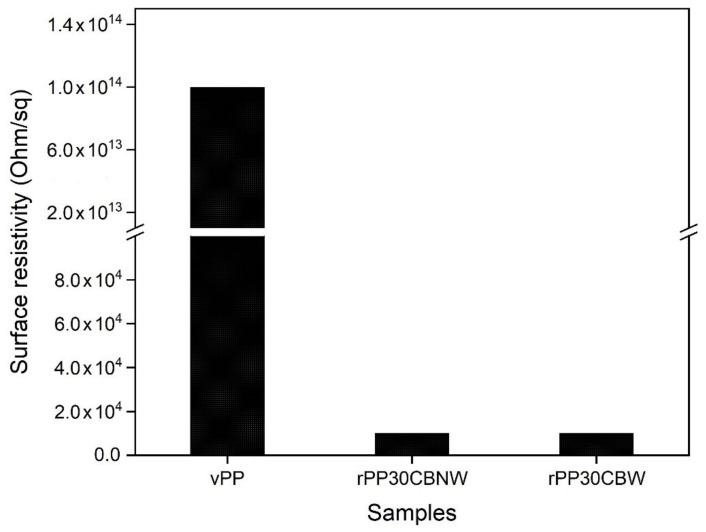
Comparison of the surface resistivity of the neat PP and the obtained rPP composites with and without the plasticizer content.

We expected these obtained composite materials to have electrical conductivity properties to use as an antistatic material. Thus, the effect of carbon black on the surface resistance of PP was studied. The surface resistivity of rPP30CB composites as a function of plasticizer content is shown in Figure 10 and also enumerated in detail in Table 3. We found that vPP displayed high surface resistivity of approximately 10^12^ Ohm/sq, while this value was reduced to 10^4^ Ohm/sq when carbon black was added into the rPP matrix. Obviously, this indicates that carbon black can improve the conductivity property of polypropylene. It is well known that PP is a natural electrical insulator with high surface resistivity. This property means that the polymer surface can resist the generation of charges [63]. We added conductive fillers such as carbon black into those materials to effectively improve this property by reducing the surface resistivity. In general, when the composite material becomes conductive enough, electrons are able to transfer from one surface to another, resulting in the electrostatic shielding property of materials [64,65]. Additionally, we found that the PE wax, used as a plasticizer for processing, is not affected by the surface resistivity of polypropylene. These results indicate that the surface resistivity of the obtained composites could be suitable for use in engineering materials applications.

### 3.5. Differential Scanning Calorimetry Test

**Figure 11:**
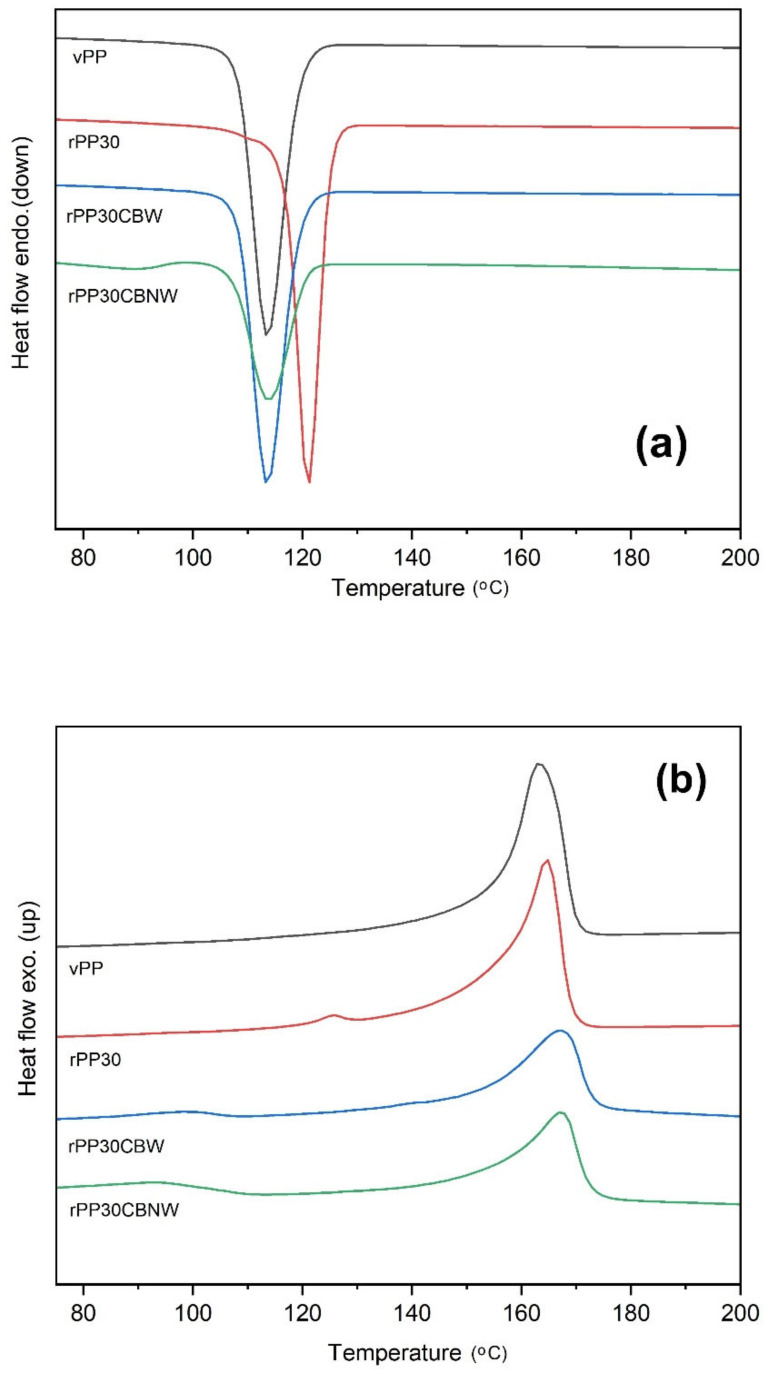
Crystallization temperature (**a**) and melting temperature (**b**) of vPP, rPP30, and its composites.

It is well known that the crystalline structure and the degree of crystallinity of polymers can affect the physical and mechanical properties of the material. Thus, DSC was performed in order to detect the crystallization and melting temperatures of all the specimens. The thermal behaviors of vPP, rPP30 and its composites are presented in Figure 11. The numerical values of the DSC thermograms were extracted and the results are summarized in Table 4. No significant difference was observed between the values measured before and after recycling for the vPP and rPP30 samples. The melting temperature of the rPP30 higher than the of vPP caused by the shorted of rPP30 can be reacted with the surface of neat PP. However, a small difference was noticed between the values of the vPP and the waste sample based on this polymer for polypropylene. Besides, it was found that rPP30 showed the small exothermic peak at 125.8 °C. This is probably attributed to admixtures of additives in the commercial waste products, which was also observed in the melting thermograms as smaller and broader curves. In addition, we found that the melting temperature peaks of rPP30 composites, both in rPP30CBNW and rPP30CBW, were higher than those of rPP30 and vPP, respectively. This result shows that the addition of carbon black particles acts as a nucleating agent, which increases the melting temperature of PP [66]. According to our investigation, in the rPP30 composites with and without PE wax (rPP30CBW and rPP30CBNW) within the rPP matrix, the melting peak temperature raised from 166.6 °C (for rPPCBW) to 167.4 °C, respectively. This indicated that the PE wax can act as a plasticizer agent, resulting in the reduction of the melting temperature of the rPP30 composite. However, the PE wax content did not show a significant influence on the thermal properties of the composites. Additionally, the small broader exothermic peaks of rPP30CBNW and rPP30CBW were found to be at 93.1 and 91.2 °C, respectively. This is probably attributed to admixtures of additives (such as carbon black and carbon black with PE wax, respectively) into the recycled composites. Interestingly, we found that the crystalline temperature enormously increased when adding rPP into the vPP matrix, as can been see in Figure 11a. rPP30 showed the highest values of crystalline temperature (approximately 121 °C) among the four samples. As we mentioned earlier, this is probably because rPP30 can be reacted with the surface of polypropylene and thus increases the crystalline temperature and the degree of crystallinity (57.10%). It was found that the degree of crystallinity increased up to approximately 26.27% when compared to the degree of crystallinity of vPP. However, in these cases, carbon black and PE wax slightly affected the crystalline temperature and the low-intensity and broader endothermic peaks were at 88.7 and 88.0 °C, respectively. The degree of crystallinity of rPP30CBNW and rPP30CBW did not significantly change and these values were calculated to be 40.36% and 39.03%, respectively. In addition, we found that the melting enthalpy of vPP was higher than that of rPP30, rPP30CBNW and rPP30CBW. As presented in Table 4, the melting enthalpy of PP decreased from 93.60 to 56.59 J/g while the fillers, such as rPP, carbon black, and PE wax, were added into PP matrices. This could indicate that the thermal stability of PP increases when adding fillers because these fillers absorb more heat energy in the melting of the composites. This finding is in agreement with the results of other literatures [67,68,69].

## 4. Conclusions

This study aimed at developing alternative upcycling polypropylene waste and antistatic composite materials with high performance and acceptable mechanical properties for various applications. The vPP/rPP blends and rPP/carbon black composites were successfully fabricated using a melt-extrusion process. All samples showed good performance of mechanical properties. However, the modulus and tensile strength of PP slightly decreased with increased recycled PP concentrations. The modulus and tensile strength of the vPP were 1317 and 36.47 MPa, respectively. These values were slightly higher than those of the vPP blended with rPP. On the other hand, the elongation at break of vPP was 15.09%. Our studies indicated that rPP can be blended with vPP at a concentration of up to 40 wt% without changing the mechanical properties significantly. In addition, it was found that MFI of the blended vPP increased with increasing rPP content.

With value-added materials in mind, we improved the electrical conductivity of recycled PP by using carbon black as a conductive filler. We found that carbon black affected the mechanical and electrical properties. The stress-strain curves of the PP composites changed from ductile to brittle behaviors. This might be caused by the poor interfacial intermolecular interaction between recycled PP and carbon black. FTIR spectra indicates that carbon black did not have any chemical reactions or bonding with the PP chains. Finally, as expected, the obtained PP-based composites exhibited good performance on the electrical properties tested. We found that vPP displayed high surface resistivity of approximately 10^12^ Ohm/sq, while this value was reduced to 10^4^ Ohm/sq when adding carbon black into rPP matrices. DSC results showed that the rPP and carbon black powder could act as nucleating agents and thus increase the degree of crystallinity of PP. The obtained rPP30/carbon black composites have potential to serve as a conductive material for various applications in the future.

## Figures and Tables

**Table 1:** Mechanical properties of the vPP specimens blended with rPP at various concentrations.

Sample Code	Modulus (MPa)	Tensile Strength (MPa)	Elongation at the Break (%)
vPP	1317 ± 55.85	36.47 ± 1.83	15.09 ± 1.75
rPP20	1252 ± 85.63	33.17 ± 2.65	16.04 ± 2.82
rPP30	1243 ± 50.15	33.75 ± 1.48	18.62 ± 1.93
rPP40	1232 ± 91.62	32.28 ± 3.12	19.72 ± 2.99

**Table 2:** Mechanical properties of rPP30, rPP30CBNW, and rPP30CBW composites.

Sample Codes	Modulus (MPa)	Tensile Strength (MPa)	Elongation at the Break (%)
rPP30	1243 ± 50.15	33.75 ± 1.48	18.62 ± 1.93
rPP30CBNW	1676 ± 87.06	17.16 ± 1.18	1.27 ± 0.08
rPP30CBW	1038 ± 22.80	26.08 ± 2.11	3.73 ± 0.47

**Table 3:** The surface resistivity of vPP, rPP30CBNW and rPP30CBW composites.

Sample Codes	Surface Resistivity (Ohm/sq)
vPP	10^12^
rPP30CBNW	10^4^
rPP30CBW	10^4^

**Table 4:** Summarized results of DSC analysis of vPP, rPP30 and its composites.

Sample Codes	*T*_c_ (°C)	*T*_m_ (°C)	$\Delta H_{m}(J/g)$	χ_c_ (%)
vPP	113.6	163.2	93.60	45.22
rPP30	121.0	164.6	82.74	57.10
rPP30CBNW	113.8	167.4	58.48	40.36
rPP30CBW	113.1	166.6	56.59	39.03

Abbreviations: PP, polypropylene; CB, carbon black; NW, non-PE wax; W, PE wax.

## Data Availability

Not applicable.

## Supplementary Materials

The following supporting information can be downloaded at: https://www.mdpi.com/article/10.3390/polym14245482/s1, Figure S1: FTIR spectra of vPP and rPP.
