# Supplementary material for: Towards a Circular Economy: Study of the Mechanical, Thermal, and Electrical Properties of Recycled Polypropylene and Their Composite Materials
Source: Polymers (Basel). 2022 Dec 14;14(24):5482. doi: 10.3390/polym14245482 (PMC9781673; doi:10.3390/polym14245482)
Supplement: Supplementary file 1 [file polymers-14-05482-s001.zip › polymers-2096339-supplementary.pdf]

polymers

MDPI

Supplementary Materials for

Towards a Circular Economy: Study of the Mechanical, Thermal, and Electrical Properties of Recycled Polypropylene and Their Composite Materials

Tongsai Jamnongkan [1*], Nitchanan Intraramongkol [1], Wesarach Samoechip [2], Pranut Potiyaraj [2], Rattanaphol Mongkholrattanasit [3], Porntip Jamnongkan [4], Piyada Wongwachirakorn [5], Masataka Sugimoto [6], Hiroshi Ito [6] and Chih-Feng Huang [7,*]

[1] Department of Fundamental Science and Physical Education, Faculty of Science at Sriracha, Kasetsart University, Chonburi 20230, Thailand
[2] Department of Materials Science, Faculty of Science, Chulalongkorn University, 10330 Bangkok, Thailand
[3] Faculty of Industrial Textiles and Fashion Design, Rajamangala University of Technology Phra Nakhon, Bangkok 10110, Thailand
[4] Department of Public Health and Environment, Saensuk Municipality, Chonburi 20130, Thailand
[5] Department of Environmental Science, Faculty of Science and Technology, Pibulsongkarm Rajabhat University, Phitsanulok 65000, Thailand
[6] Graduated School of Organic Materials Science, Faculty of Engineering, Yamagata University, Yonezawa 32000, Yamagata, Japan
[7] Department of Chemical Engineering, i-Center for Advanced Science and Technology (*i*CAST), National Chung Hsing University, Taichung 40227, Taiwan
* Correspondence: jamnongkan.t@ku.ac.th (T.J.); huangcf@dragon.nchu.edu.tw (C.-F.H.)

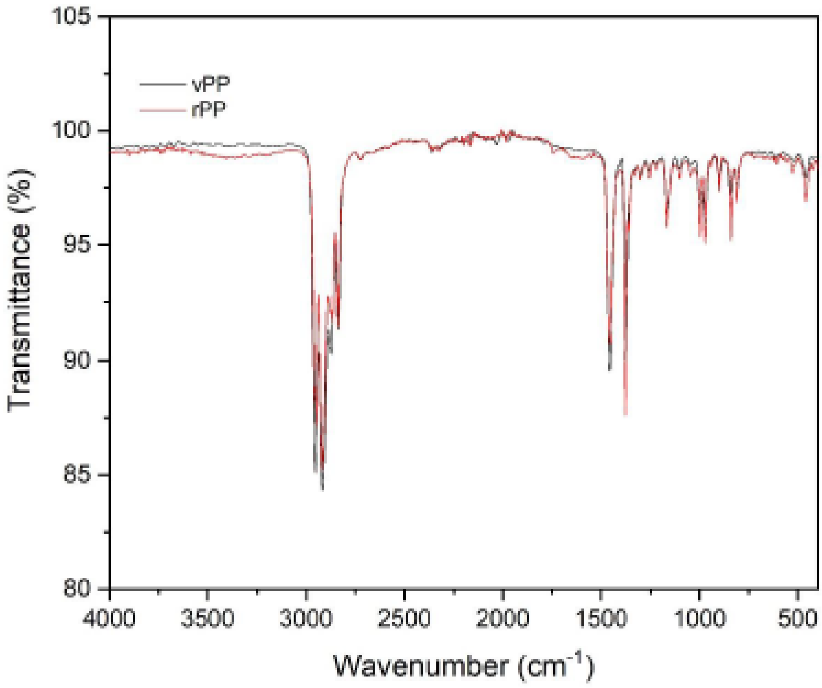

Figure S1. FTIR spectra of vPP and rPP.
